# Programmable Digital-Microfluidic Biochips for SARS-CoV-2 Detection

**DOI:** 10.3390/bioengineering10080923

**Published:** 2023-08-03

**Authors:** Yuxin Wang, Yun-Sheng Chan, Matthew Chae, Donglu Shi, Chen-Yi Lee, Jiajie Diao

**Affiliations:** 1 Department of Cancer Biology, University of Cincinnati College of Medicine, Cincinnati, OH 45267, USA; 2 The Materials Science and Engineering Program, Department of Mechanical and Materials Engineering, College of Engineering and Applied Science, University of Cincinnati, Cincinnati, OH 45221, USA; 3 Advanced Sensing Lab, Digital Futures, University of Cincinnati, Cincinnati, OH 45221, USA; 4 Institute of Electronics, National Yang Ming Chiao Tung University, Hsinchu 30010, Taiwan

**Keywords:** digital-microfluidic biochip (DMFB), micro-electrode-dot-array (MEDA), programmable biochip, loop-mediated isothermal amplification (LAMP), SARS-CoV-2

## Abstract

Biochips, a novel technology in the field of biomolecular analysis, offer a promising alternative to conventional testing equipment. These chips integrate multiple functions within a single system, providing a compact and efficient solution for various testing needs. For biochips, a pattern-control micro-electrode-dot-array (MEDA) is a new, universally viable design that can replace microchannels and other micro-components. In a Micro Electrode Dot Array (MEDA), each electrode can be programmatically controlled or dynamically grouped, allowing a single chip to fulfill the diverse requirements of different tests. This capability not only enhances flexibility, but also contributes to cost reduction by eliminating the need for multiple specialized chips. In this paper, we present a visible biochip testing system for tracking the entire testing process in real time, and describe our application of the system to detect SARS-CoV-2.

## 1. Introduction

Microfluidic biochips (MFBs), commonly referred to as “labs-on-a-chip,” have revolutionized the field of biomolecular analysis, particularly in high-throughput DNA sequencing and point-of-care clinical diagnosis [1,2]. These biochips utilize the principle of electro-wetting-on-dielectric (EWOD) [3], and have overcome various limitations associated with traditional flow-based MFBs, such as size and defect tolerance. One significant advancement in MFB technology is the development of Digital-MicroFluidic Biochips (DMFBs) [4]. DMFBs employ EWOD principles and offer the ability to integrate multiple functions on a single chip. Li et al. [5] introduced a micro-electrode-dot-array (MEDA) for novel DMFBs, which comprises thousands of equally sized electrodes. These electrodes can be independently controlled or coordinated in groups to perform various operations dynamically [6,7,8]. The field of biochips has witnessed notable progress, and several representative biochips have been developed for specific biological tests. For instance, high-throughput DNA sequencing has been made feasible with the use of biochips like Illumina’s HiSeq and MiSeq systems [9,10]. These biochips enable the parallel processing of millions of DNA fragments, significantly accelerating the sequencing process. Biochips have also found applications in point-of-care clinical diagnosis. The development of microfluidic platforms, such as the GeneXpert system from Cepheid, has allowed for rapid and accurate detection of infectious diseases, including tuberculosis and respiratory infections [11]. These biochips integrate sample preparation, nucleic acid amplification, and detection steps, enabling efficient diagnosis in resource-limited settings. Thus, the field of biochips has experienced significant advancements through the development of DMFBs and their integration of multiple functions on a single chip. Representative biochips, such as those used in high-throughput DNA sequencing and point-of-care clinical diagnosis, have demonstrated the potential of these technologies in transforming biomolecular analysis. Programmable DMFBs with Adaptive Pattern-Control Micro-Electrode-Dot-Array (PC-MEDA) represent a significant advancement in biochip technology. Unlike traditional biochips, PC-MEDA-based DMFBs offer enhanced flexibility, adaptability, and control in performing various bioanalytical operations. PC-MEDA allows for the dynamic coordination of micro-electrodes in the biochip, enabling precise manipulation of droplets and efficient execution of complex bioassays. This programmable capability enables on-demand reconfiguration of electrode patterns, optimizing the chip’s performance for different applications [5]. The adaptive pattern-control feature of PC-MEDA-based DMFBs addresses the limitations of fixed electrode layouts in traditional biochips. It allows for the customization of electrode patterns to match specific assay requirements, facilitating efficient fluidic routing, droplet splitting, merging, and dispensing operations [5,6]. The programmability and adaptability of PC-MEDA-based DMFBs have opened up new avenues for biochip applications. One notable application is in the field of high-throughput drug screening, where PC-MEDA enables precise control of droplet manipulation and reagent delivery, enhancing the efficiency and accuracy of screening processes [7]. Another promising application is in the development of point-of-care diagnostic devices. PC-MEDA-based DMFBs offer the capability to perform multiplexed assays, where different biomarkers can be simultaneously analyzed on a single chip. This technology has the potential to revolutionize rapid and accurate diagnosis of infectious diseases and other medical conditions [7,8].

Reverse Transcription Loop-Mediated Isothermal Amplification (RT-LAMP) is a variant of the LAMP method that is specifically designed for the amplification of RNA sequences. It combines the principles of reverse transcription and LAMP to enable the detection of RNA viruses and other RNA sequences [12,13,14,15]. In the RT-LAMP process, the RNA target sequence is first reverse-transcribed into complementary DNA (cDNA) by a reverse transcriptase enzyme. This cDNA then serves as the template for the LAMP reaction. The LAMP reaction involves the use of a DNA polymerase and a set of four to six specially designed primers that recognize six to eight distinct regions on the target DNA. The reaction proceeds at a constant temperature, typically in the range of 60–65 degrees Celsius, and results in the amplification of the target sequence. The RT-LAMP method is highly sensitive and specific, capable of amplifying very small amounts of target RNA with high efficiency. It is also rapid, with results typically available within an hour. Because it does not require the temperature cycling of traditional Polymerase Chain Reaction (PCR) methods, it can be performed using simple and inexpensive equipment, making it suitable for use in point-of-care and field settings. This is a method of RNA amplification for targeting genes which have been used to detect SARS-CoV-2 [16]. The detection methods for LAMP reaction products include electrophoresis, turbidity, and dye methods. Among them, electrophoresis and turbidity are endpoint detection methods, while dye methods are now more commonly used for real-time detection. 

The dyes used in LAMP dye methods include self-luminous fluorescent dyes, intercalating fluorescent dye, and colorimetric dyes (pH indicators). Common self-luminous fluorescent dyes include Hydroxynaphthol Blue (HNB), Calcein, and so on [17,18,19]. HNB is a metal ion indicator. When HNB binds with Mg^2+^, the initial color of the reaction system is purple. As the reaction proceeds, Mg^2+^ reacts with the precipitated P_2_O_7_^4−^ to form Mg_2_P_2_O_7_ precipitate. HNB loses Mg^2+^, causing the system color to change to sky blue, while the unreacted system remains purple. Calcein requires both additional Mg^2+^ and Mn^2+^ in the system. As a chelating agent, Calcein binds with Mn^2+^ in a quenched state, not showing fluorescence. During the LAMP reaction, the generated P_2_O_7_^4−^ will bind with Mn^2+^ to relieve the quenching state, and free Calcein will bind with Mg^2+^ and emit yellow-green fluorescence. Common intercalating fluorescent dyes include SYBR Green, EvaGreen, and SYTO [20,21]. These dyes infiltrate into the minor grooves of DNA double strands. After binding with double-stranded DNA, the fluorescence signal can be enhanced 800 to 1000 times. The fluorescent dye method can achieve real-time and visual detection when using a special designed heating block with a fluorophotometer, suitable for detections requiring high sensitivity. The last one is the colorimetric dye method. During the LAMP reaction, the by-products released during synthesis include H^+^ and P_2_O_7_^4−^, so the pH of the reaction solution changes from initially alkaline to acidic as the reaction proceeds. This significant pH change provides another possibility for detecting LAMP reaction by using pH-sensitive indicators, which allows the visual judgment of amplification results and distinguishing between positive and negative results. The reaction using pH indicators as dyes requires low buffer capacity (low concentration Tris), with a pH above 8.8. The method makes low-cost, portable rapid LAMP detection easier to implement and popularize. The common pH indicators for LAMP test include phenol red and cresol red. Both of them will show a color change from red to yellow when amplification occurs [22].

According to recent studies on LAMP, colorimetric dyes are becoming the most popular choice. Several papers have reported using this technique to obtain visible results of SARS-CoV-2 detection [16,23,24,25]. Meanwhile, other research has indicated that crude samples in a clinical setting can be used in the LAMP tests without RNA purification, which would not restrain the specific amplification reaction [16,26,27]. However, uncertainties in the pH values of crude samples often result in the solution turning orange. This also occurs with the low-concentration positive samples. Therefore, enhanced procedural controls with pretreated pH values have been proposed and tested for the optimization of components by adding MgSO_4_ [28]. Mg^2+^ can be expected to react with the P_2_O_7_^4−^ by-products to promote amplification while yielding the white precipitate of the Mg_2_P_2_O_7_ [18]. The white precipitate can be regarded as another sign of amplification, and this is called the turbidity method. As mentioned before, this method has been classified to the endpoint methods because the precipitate cannot be observed as clearly as the change in color with a small volume of LAMP testing samples, and a turbidimeter will be needed to determine the turbidity change after the reaction takes place. 

To address these critical issues, we employed programmable PC-MEDA-based DMFBs [29] in the RT-LAMP testing to detect SARS-CoV-2 according to a visible change in turbidity. Through this method, the endpoint turbidity detection changed to a real-time visible indicator of LAMP reaction. On the biochips, the white precipitate of Mg_2_P_2_O_7_ was hypothesized to indicate a change in turbidity in the sample. We developed a real-time imaging system capable of monitoring the entire process of LAMP reactions. 

## 2. Experimental Setup

### 2.1. Materials

2× WarmStart LAMP Master Mix (WarmStart LAMP Kit (DNA & RNA), #E1700S, New England Biolabs, Ipswich, MA, USA), WarmStart Colorimetric LAMP 2× Master Mix (WarmStart Colorimetric LAMP 2× Master Mix (DNA & RNA), #M1800S, New England Biolabs, Ipswich, MA, USA), 5× LAMP Buffer Mix, 10× Enzyme Mix, 5× SARS-CoV-2 Primer Mix (Invitrogen Colorimetric ReadiLAMP Kit, SARS-CoV-2, Fisher Scientific, Hampton, NH, USA), AcroMetrix SARS-CoV-2 Control (Full-process) (Thermo Scientific, Waltham, MA, USA), Ethidium bromide stock solution (VWR), Molecular Biology Grade Water (Fisher Scientific), 1 kb DNA ladder (#N3232S, New England Biolabs, Ipswich, MA, USA).

### 2.2. LAMP Reagents

A mix of 12.5 μL 2× LAMP Master Mix, 5 μL 5× SARS-CoV-2 Primer Mix for WarmStart LAMP Kit, 5 μL 5× LAMP Buffer Mix, 2.5 μL 10× Enzyme Mix, 5 μL 5× SARS-CoV-2 Primer Mix for Invitrogen Colorimetric ReadiLAMP Kit, and 2 μL AcroMetrix SARS-CoV-2 Control (Thermo Scientific, Waltham, MA, USA) was used as the positive control sample, whereas the same volume of nuclease-free water was used for the negative control. The testing solution was filled with nuclease-free water until a final volume of 25 μL was obtained. Because the SARS-CoV-2 Primer Mix contains Mg^2+^, no additional chemicals were added to the samples.

### 2.3. LAMP Test

To initiate the experiment, a volume of 1.5 μL from the mixed LAMP solution was carefully applied onto the biochips, which were then covered with Indium tin oxide (ITO) glass. The droplet on the biochips was surrounded by silicon oil. The remaining 23.5 μL of the solution was placed in polymerase chain reaction (PCR) tubes for further processing. The biochips were subjected to a temperature of 65 °C for a duration of 30 min in the testing area. Throughout the procedure, the microscope was employed to observe and track the entire process, which was also recorded using video. For the remaining sample in the PCR tubes, a Programmable Thermal Controller PTC-100 (MJ Research Inc., Waltham, MA, USA) was utilized. The incubation temperature was set to 65 °C, and the tubes were incubated for 30 min.

To assess the end-point results and detect nucleic acid amplification in the tube samples, a 1% agarose gel containing 0.5 μg/mL ethidium bromide (EB) was employed.

### 2.4. Biochip System

**Figure 1:**
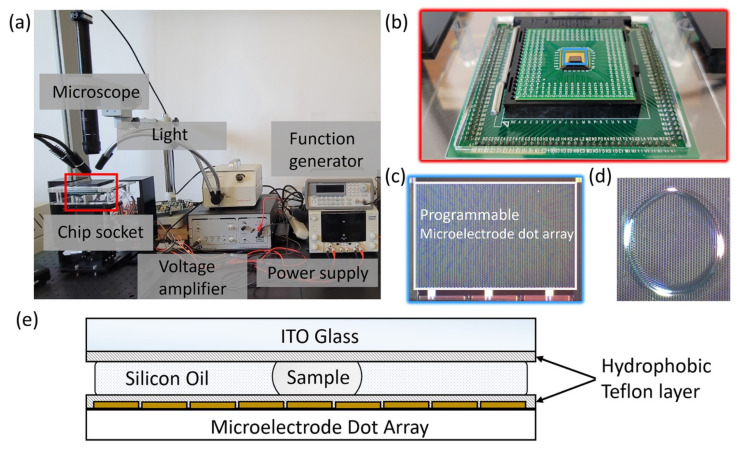
(**a**) DMFB bio-test platform with real-time observation system. (**b**) Chip socket detail of red box in Figure 1a with a chip. (**c**) Microscope imaging of sample loaded area on the chip (the detail image of blue box in Figure 1b). (**d**) Microscope imaging of a droplet sample on the chip. (**e**) Sandwich structure of testing environment.

The PC-MEDA biochip, as depicted in Figure 1c, is a sophisticated piece of technology that has been implemented using standard 0.35 μm 2 poly/4 metal complementary metal-oxide-semiconductor (2P4M CMOS) technology. Compared with previous biochips [6,30,31,32], our chips have a smaller electrode size, large number of electrodes, lower power usage, and TiN/AI/TiN materials. All of the above make the chip show a higher integration performance. This biochip is a marvel of miniaturization, containing a staggering 5400 micro-electrodes (MEs). These MEs are not just simple electrical contacts, but are designed to facilitate digital microfluidic operations and temperature control. This is achieved through the use of specific 2D binary patterns, which are essentially digital instructions that guide the behavior of the biochip.

The materials used in the construction of the PC-MEDA biochip are carefully selected to ensure optimal performance. The biochip itself is fabricated from a CMOS substrate; a material that is widely used in the manufacture of integrated circuits due to its excellent electrical properties. The micro-electrodes are made from a conductive material that allows for the precise control of electrical signals. The biochip also includes an ITO glass plate, which is filled with silicon oil. This plate is crucial for the operation of the biochip, as it is used to sandwich the sample or droplet that is being analyzed.

The fabrication process of the PC-MEDA biochip involves several steps. First, the CMOS substrate is prepared, and the micro-electrodes are patterned onto it using photolithography, a process that allows for the creation of extremely small and precise structures. Then, the capacitive sensing circuits are integrated into each micro-electrode. These circuits are crucial for the operation of the biochip, as they allow for the identification of the location and size of the target samples. Finally, the ITO glass plate is attached to the biochip, completing the assembly process.

The PC-MEDA biochip operates using a pattern-control mechanism. This mechanism uses 2D binary patterns to manipulate microfluidic processes and temperature profiles. The biochip employs EWOD-based digital microfluidics, a technique that uses electric fields to manipulate droplets of liquid. The droplet or sample is sandwiched between the biochip and the ITO glass plate, and a 1 kHz 50 Vp-p square wave is applied to the top ITO glass plate. This generates the necessary driving force to move the droplets or samples towards the actuated MEs. Furthermore, the PC-MEDA biochip is not just a passive device, but is capable of real-time feedback control. This is achieved through the use of the built-in capacitive sensing circuits, which monitor the position and size of the droplets or samples. This information is then used to adjust the 2D binary patterns, ensuring that the droplets or samples follow their designated routes. In addition to its microfluidic capabilities, the PC-MEDA biochip also serves as a micro-heating array. This is achieved by applying different 2D binary patterns to the MEs, which allows for the precise control of temperature profiles. The temperature can be digitally controlled, making it possible to achieve precise control over the density of actuated MEs. 

In the suggested mechanism for pattern control, the ME serves as a fundamental component or a basic unit for constructing the entire system. Our design incorporates three functional modules on the proposed biochip: digital microfluidics; temperature profiling; and capacitive sensing. Each ME is designed to operate and be programmed independently. A double-layer structure is employed in the electrode design to facilitate multi-functionality in each ME. The first layer (top layer) is dedicated to microfluidic operations and capacitive sensing, while the second layer is utilized for temperature profiling and acts as a shielding layer for microfluidic operations and capacitive sensing. The Joule heating method is leveraged to create varying temperature profiles on the proposed biochip. To implement the pattern-control mechanism, a standard hot plate is divided into smaller sections and incorporated into each ME. The on/off switch on each ME operates independently from other MEs. Consequently, the temperature control circuit on each ME is triggered by an input 2D binary pattern. The heating current is regulated by the resistor RHEAT, which is approximately 1 mA in each ME design. A zigzag structure is employed in the heating electrode design on the top layer to achieve a more uniform temperature distribution. To prevent high currents from passing through the digital circuit on MEs, the supply voltage for heating is isolated from the supply voltage for digital circuits, and can be individually controlled.

Figure 1a,b illustrates the measurement environment and the chip socket, respectively. In the experimental setup, the sample is sandwiched between the ITO glass (top) and the PC-MEDA biochip (bottom), as depicted in Figure 1d,e. Teflon is used to create a hydrophobic layer on top of the ITO glass and the PC-MEDA biochip, while silicon oil is used to prevent the evaporation of samples during experimental runs.

## 3. Results and Discussion

**Figure 2:**
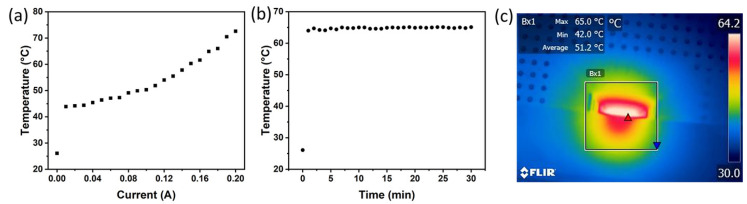
(**a**) Temperature vs. current curve. (**b**) Temperature vs. time curve under LAMP test mode. (**c**) Thermal images of testing area at LAMP test mode.

Prior to conducting the bio-tests, the temperature was measured. A thermal imaging system (FLIR ETS320, Teledyne FLIR LLC) was used to monitor the temperature of the sample-loaded area, as exemplified in Figure 2c. The entire MEDA is programmed as a heating plate, and the heating temperature is controlled by the current passing through the electrodes. All the measurements have been done at room temperature with both aqueous droplets and silicon oil loaded on the chip and the imaging assist light on. Figure 2a displays the temperature versus current curve, while Figure 2b presents the real-time temperature during the LAMP test mode. The current for heating to 65 °C is around 0.18 A, but it is not the upper limit of the current. The temperature can be adjusted in a wide range, which shows potential to replace both traditional water baths and heating blocks when handling a small amount of the liquid sample to reduce energy waste. The best temperature for the LAMP test is from 60–65 °C; through the measurement, the heating area can reach the testing temperature in 2 min and remain stable. A slight vibration showed in Figure 2b for 30 min of temperature tracking, but all stay in the requirement range. Even after 30 min of heating, at a much higher temperature stage than at the start, the constant current can be used, which means the materials of electrodes can keep a stable resistance under different conditions. 

**Figure 3:**
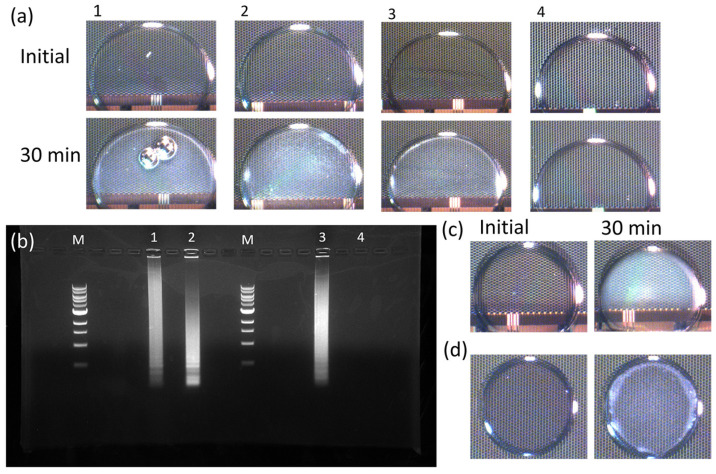
(**a**) Starting-point and end-point images of RT-LAMP samples with a standard heating process (65 °C 30 min) on chips. Samples 1–3 are positive samples, and Sample 4 is a negative control. (**b**) End-point Agarose gel results (stained by Ethidium bromide), lanes: M 1 kb DNA ladder, 1–4 the same samples corresponding to Samples 1–4 in (**a**). Visible turbidity changes of colorimetric LAMP samples on chips for (**c**) Phenol red and (**d**) Hydroxy naphthol blue.

The microscope-based records of chip tests depicted in Figure 3a exhibit four samples, all of which were created using the transparent solution, New England Biolabs 1700M LAMP Mix. The test solution was prepared following LAMP reagents as mentioned before. A 25 μL solution was placed in a tube for all positive and negative samples. A 1.5 μL sample was loaded between a chip and a slice of ITO glass with extra silicon oil surrounding the droplet. The droplets were observed to be transparent in all four initial images, and the electrode dot array can be seen clearly as the background at the initial imaging of all samples. Following the heating cycle, Samples 1–3 exhibited a noticeable white, cloudy appearance, while Sample 4 retained its transparent nature. The cloud is the Mg_2_P_2_O_7_ white precipitate, which is generated by amplification reaction. Thus, the results indicate that an amplification reaction occurred in Samples 1–3, indicating a positive detection for SARS-CoV-2. In contrast, Sample 4 did not show any amplification, indicating a negative result for SARS-CoV-2.

To confirm the reliability of the findings, the remaining samples were subjected to incubation using a thermal controller, followed by an end-point agarose gel test. The agarose gel test, also known as agarose gel electrophoresis, is a common laboratory technique used to separate DNA, RNA, or protein molecules based on their size and charge. By applying an electric field, these molecules are made to move through an agarose matrix, and smaller molecules move faster than larger ones, allowing for their separation and subsequent analysis. This is a reliable endpoint method to analyze LAMP results. The feature of LAMP is that the produced gene fragments have various sizes, and the number of fragments is as high as 10^9^ times the original reagent. The added EB in the gel is an intercalator, and when this meets a double helix, it will insert between the base pairs and generate fluorescent property. The result of the gel will read under UV light to see if any fluorescent signal is present. So, if amplification happened, the gel result will show a continuous bright pattern. On the other side, the negative result should show no signal, or show a very weak signal on the bottom of the lane due to the sticky primers. However, it is good for research use but not fit for point-of-care testing. 

Figure 3b exhibits the gel image showcasing the remaining portions of Samples 1–4 subsequent to their incubation using a heating block. Lane M is the DNA ladder, while Lanes 1–4 correspond to Samples 1–4. The gel image shows distinct ladder-like patterns in Lanes 1–3, indicating positive results. In contrast, the absence of such a pattern in Lane 4 suggests a negative result. Thus, the gel-based results are consistent with the biochip-based results. Furthermore, in order to verify the universal applicability of biochips in LAMP testing, the colorimetric LAMP Mix was employed to assess if the change in turbidity can still be observed. Figure 3c exhibits images of the sample containing phenol red, displaying pink droplets prior to heating, which transitions to yellow if the result is positive. On the other hand, Figure 3d shows the sample containing hydroxy naphthol blue, demonstrating purple droplets before heating, which changes to blue in the event of a positive result. In both cases, the samples exhibited a noticeable increase in cloudiness when amplification occurred. Thus, the change in turbidity can be considered a robust signal in chip imaging that remains unaffected by the original color of the testing samples. The imaging system in this study was set up to meet the requirement of replacing turbidimeter to make the result visible to the naked eye, and change the endpoint method to being observable in real time, so the color change cannot be distinguished on chips. The imaging system is independent to the chip system. It is not specially designed for this chip system, and can be changed to fit other requirements in further use.

## 4. Conclusions

We developed a bio-testing system incorporating an imaging subassembly, which utilized programmable digital-microfluidic biochips with a MEDA (Micro Electrode Dot Array) platform. The system was utilized to conduct LAMP tests, and with the assistance of a microscope-based imaging system, the entire LAMP reaction process could be visualized and recorded. A change in turbidity was observed when nucleic acid amplification occurred. In contrast, it is often challenging to discern a reliable signal during tests when reactions take place in a tube format. Thus, the colorimetric LAMP buffer was designed to obtain visible results, or additional fluorescent dye was added to normal LAMP buffer as a marker. Nevertheless, in practical applications, additional steps are typically required to manipulate raw samples, such as amplifying the targeted genes or adjusting the pH to specific levels. These extra steps not only prolong the testing time, but also contribute to increased costs. Although most LAMP reagents were introduced as being observable in real time, it is impossible to view the process when samples are incubated in a heating block, unless expensive equipment with a spectra detector is available. For the chip system, the sample volume required is less than one-tenth of what is needed in traditional LAMP tests, and the visible feature does not rely on the change in color, which means fewer restrictions in testing raw samples. Furthermore, real-time tracking enables accurate monitoring of the actual implementation of the amplification reaction on the chips. It changed the fact that the turbidity method can only be an endpoint method. Additionally, the imaging component can be easily modified by replacing it with different microscopes or light sources, allowing for higher-resolution imaging or the observation of color changes as required. In conclusion, the programmable control system has the capacity to support more intricate heating cycles for various tests, including PCR. This is a successful try of using our biochip system to a real biotest, which will be a foundation to design a new generation of biochips and a more flexible system. In future investigations, it is crucial to design and evaluate additional bio-protocols utilizing biochips, expanding the potential applications of this technology. In future investigations, we can further improve the portability and flexibility of the platform and imaging, such as adding more operable remote settings and using low-cost miniature lensless imaging setups [33]. The power supply for this chip is 3.3 V, and in future, the chips can be designed like a flash drive, which uses a common charger to replace the existing power supply. 

## Data Availability

Not applicable.
